# Pilot trial testing the effects of exercise on chemotherapy-induced peripheral neurotoxicity (CIPN) and the interoceptive brain system

**DOI:** 10.21203/rs.3.rs-4022351/v1

**Published:** 2024-03-13

**Authors:** Ian R. Kleckner, Thushini Manuweera, Po-Ju Lin, Kaitlin H. Chung, Amber S. Kleckner, Jennifer S. Gewandter, Eva Culakova, Madalina E. Tivarus, Richard F. Dunne, Kah Poh Loh, Nimish A. Mohile, Shelli R. Kesler, Karen M. Mustian

**Affiliations:** University of Maryland School of Nursing; University of Maryland School of Nursing; University of Rochester Medical Center; Cornell University; University of Maryland School of Nursing; University of Rochester Medical Center; University of Rochester Medical Center; University of Rochester Medical Center; University of Rochester Medical Center; University of Rochester Medical Center; University of Rochester Medical Center; The University of Texas at Austin; University of Rochester Medical Center

**Keywords:** chemotherapy-induced peripheral neurotoxicity (CIPN), neuropathy, exercise, interoception, brain, fMRI

## Abstract

**Purpose.:**

Chemotherapy-induced peripheral neurotoxicity (CIPN) is a prevalent, dose-limiting, tough-to-treat toxicity involving numbness, tingling, and pain in the extremities with enigmatic pathophysiology. This randomized controlled pilot study explored the feasibility and preliminary efficacy of exercise during chemotherapy on CIPN and the role of the interoceptive brain system, which processes bodily sensations.

**Methods.:**

Nineteen patients (65±11 years old, 52% women; cancer type: breast, gastrointestinal, multiple myeloma) starting neurotoxic chemotherapy were randomized to 12 weeks of exercise (home-based, individually tailored, moderate intensity, progressive walking and resistance training) or active control (nutrition education). At pre-, mid-, and post-intervention, we assessed CIPN symptoms (primary clinical outcome: CIPN-20), CIPN signs (tactile sensitivity using monofilaments), and physical function (leg strength). At pre- and post-intervention, we used task-free (“resting”) fMRI to assess functional connectivity in the interoceptive brain system, involving the salience and default mode networks.

**Results.:**

The study was feasible (74–89% complete data across measures) and acceptable (95% retention). We observed moderate/large beneficial effects of exercise on CIPN symptoms (CIPN-20, 0–100 scale: −7.9±5.7, effect size [ES]=−0.9 at mid-intervention; −4.8±7.3, −ES=0.5 at post-intervention), CIPN signs (ES=−1.0 and −0.1), and physical function (ES=0.4 and 0.3). Patients with worse CIPN after neurotoxic chemotherapy had lower functional connectivity within the default mode network (R^2^=40–60%) and higher functional connectivity within the salience network (R^2^=20–40%). Exercise tended to increase hypoconnectivity and decrease hyperconnectivity seen in CIPN (R^2^ = 12%).

**Conclusion.:**

Exercise during neurotoxic chemotherapy is feasible and may attenuate CIPN symptoms and signs, perhaps via changes in interoceptive brain circuitry. Future work should test for replication with larger samples. ClinicalTrials.gov identifier NCT03021174.

## Introduction

Two-thirds of patients receiving taxane, vinca alkaloid, platinum agent, bortezomib, or thalidomide-based chemotherapy develop chemotherapy-induced peripheral neurotoxicity (CIPN) [1]. CIPN is a dose-limiting toxicity [2] and can involve numbness, tingling, pain, cold allodynia, cramping, and weakness in the hands and feet. It can compromise balance and walking and increase risk of falls [3]. CIPN can also reduce a patient’s quality of life by interfering with daily activities such as dressing, typing, and writing. Despite over 20 years of research and over 100 clinical trials, there are limited options for preventing or treating CIPN [4–6]. The drug duloxetine reduces pain in CIPN [4] but its side effects [7] and low uptake [8] suggest the need for more treatment options.

**Figure 1:**
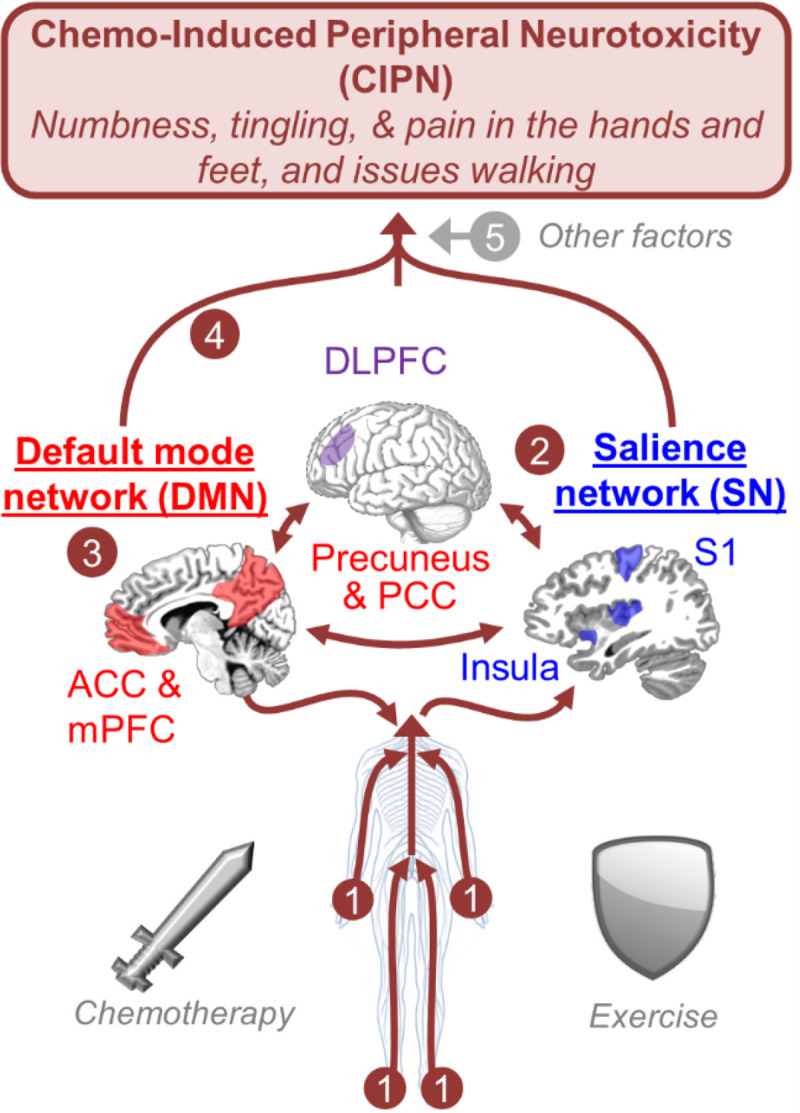
Our theoretical framework that CIPN symptoms are a combined result of (1) peripheral nerve damage, (2, 3, 4) changes in key brain networks that support interoception [24]—the processing of sensations from the hands, feet, and other areas of the body, and (5) other factors (e.g., inflammation and catastrophizing, discussed elsewhere [16]), and that exercise can attenuate CIPN symptoms by protecting against those changes [14, 16]. (1) Chemotherapy-induced peripheral nerve damage yields hyperactivity (contributing to pain) and axonal loss (contributing to numbness). This alters the type and frequency of peripheral signals sent to the central nervous system. (2) The salience network (SN; blue [39]) receives these altered peripheral signals via projections through the spinal cord to the brain (thalamus, S1, insula, etc.). The brain alters its activity and connectivity in response to this altered input, namely with increased resting activity (per our review [14] and published data on S1 reactivity when patients focus attention on their symptoms [15]). (3) The default mode network (DMN; red [40]) helps to integrate and interpret the peripheral signals relayed through the SN to the anterior cingulate cortex (ACC), medial prefrontal cortex (mPFC), precuneus, and posterior cingulate cortex (PCC). These regions become hyperactive and must change in the context of CIPN because many of the sensory inputs are unexpected [41–43] (i.e., unexpectedly *not*feeling something when you touch it or unexpected burning/shooting pain *without*touching anything). The DMN also hypothetically generates a model of the body and is central to initiating perceptions [24], including symptoms of CIPN. (4) Dorsolateral prefrontal cortex (DLPFC; purple [28]). The DLPFC is part of several networks and supports attention to body sensations including those experienced as pain [28]. In a healthy system, the DLPFC can help inhibit pain, partly by “descending modulation,” inhibiting activity of regions such as S1 and the precuneus [44–46]. The manner in which the brain changes during neurotoxic chemotherapy may be critical for determining whether CIPN symptoms become severe and chronic, like brain reorganization seen in chronic pain [46]and stroke [47]. Thus, we hypothesize that CIPN is exacerbated by interaction between regions within and across the DMN, SN, and DLPFC, and these interactions can be assessed non-invasively using functional connectivity fMRI [48]. Finally, we hypothesize that exercise can protect the brain against these changes, as exercise has been shown to increase connectivity within the DMN in older adults [49].

Exercise is emerging as a promising and safe intervention for CIPN [9–12]. In a 2018 exploratory analysis conducted with 355 patients, we found that six weeks of progressive walking and resistance training may alleviate CIPN symptoms of hot/coldness in the hands/feet and numbness and tingling [13]. We followed up on our results with this pilot study testing the same Exercise for Cancer Patients^®©^ (EXCAP) intervention for twice the duration along with more rigorous CIPN assessments, a time- and attention-matched control condition, and fMRI to study the role of the brain in CIPN. Indeed, while most mechanistic research on CIPN has focused on peripheral nerve damage, our recent work outlines our hypothesis and supporting evidence that the brain may be involved in CIPN [14, 15] (Figure 1), even if chemotherapy does not enter the brain. Ultimately, we aim to apply mechanistic measures of the brain to improve the prediction, prevention, and treatment of CIPN [14, 15] and to optimize potential benefits of exercise on CIPN [16].

We designed this pilot study to examine three hypotheses: (1) it is feasible to test the effects of exercise on CIPN and the interoceptive brain system using fMRI, (2) exercise during neurotoxic chemotherapy alleviates CIPN, and (3) the interoceptive brain system is related to CIPN and is affected by exercise.

## Methods

See the Supplementary Information for detailed methods.

### Study design.

This is a Phase I/II pilot feasibility randomized controlled trial (RCT) pre-registered at clinicaltrials.gov (NCT03021174) to assess the feasibility and preliminary efficacy of exercise vs. a behavioral placebo control on CIPN and the interoceptive brain system. We obtained ethical approval (RSRB 66046) in accordance with the Nuremberg Code, the Declaration of Helsinki, and the Belmont Report. We collected data at University of Rochester Medical Center in Rochester, NY, USA from 2017–2018. All participants provided written informed consent before participation.

Participants were ≥18 years of age, had cancer, had at least six months life expectancy, were scheduled to receive neurotoxic chemotherapy (taxanes, platinums, vinca alkaloids, thalidomide, or bortezomib), were safe for brain MRI, were safe to exercise, and were not exercising more than two days per week according to exercise stages of change [17]; exercise was defined as aerobic or strength training for at least 20 min/session at an intensity that increases heart rate, breathing, and causes sweating.

Participants were randomized 1:1 to exercise or a time- and attention-matched control (nutrition education), stratified by severity of numbness/tingling in the hands/feet (rated 0–4 vs. 5–10) and chemotherapy type (taxane vs. other). A statistician not involved in recruitment (EC) made the randomization table using blocks of 2 or 4 and it was concealed from the researchers. Randomization occurred after completion of baseline assessments.

### Outcome measures (Supplementary Figure 1).

There were three assessment time points: pre-intervention was typically 1 week before the first dose of neurotoxic chemotherapy, mid-intervention was 6–7 weeks after starting neurotoxic chemotherapy and the intervention, and post-intervention was 12–13 weeks after starting neurotoxic chemotherapy. Each time point included several assessments: (1) Patient-reported questionnaires including the primary clinical outcome, the CIPN-20 [18] scaled 0–100 (higher is worse) [19] and a symptom inventory. (2) CIPN signs assessed via tactile sensitivity test on the pointer finger and great toe using monofilaments. (3) Physical function tests including 6-min walk test, handgrip dynamometry (Jamar), and isokinetic leg strength (Biodex System 4; Biodex Medical Systems, Inc., Shirley, NY). (4) Daily steps using a wrist-worn Garmin Vivofit activity tracker for 3–14 days at pre-, mid-, and post-intervention (exercisers wore the device throughout the study). (5) Brain MRI at pre- and post-intervention on a Siemens 3T MRI MAGNETOM PrismaFit scanner with a 7.5-min task-free (“resting”) T2-weighted functional MRI scan, and a novel 8-min bodily attention task (results in [15]). (6) Interviews at the end of the study assessed acceptability.

### Exercise intervention.

The 12-week EXCAP intervention is a home-based, individually tailored, moderate-intensity walking and resistance exercise program described previously [13] and in detail in Supplementary Materials. It includes a 1-hour session with an American College of Sports Medicine (ACSM)-certified exercise professional, a book describing the intervention, three TheraBand resistance bands (red, green, and blue), and a wrist-worn activity tracker (Garmin Vivofit). Daily walking is moderate intensity (rating of perceived exertion; RPE 5–8/10), with daily steps goals based on pre-intervention average daily steps and weekly progression of 5–20% (patient’s choice). The 16 resistance exercises were to be performed daily at RPE 5–8/10 progression throughout the entire 12-week intervention period up to 4 sets of 15 repetitions.

### Control condition.

The 12-week control condition was a nutrition education program designed to match the exercise intervention in time, attention, and expectation of benefit [20]. The control condition included a 1-hour instructional session by the principal investigator (IRK) and a book by the National Cancer Institute (NCI) called Eating Hints: Before, During, and After Cancer Treatment. The control instructional session started with a discussion of the participant’s eating habits, choosing foods to manage chemotherapy toxicities, and recommendations to eat 5 servings of fruits or vegetables per day if that was safe and feasible (e.g., 3 fruits plus 2 vegetables).

### Matching conditions.

We designed nutrition education to match the exercise intervention in several ways: (1) similar time and effort (1 hour initial meeting and daily homework with follow-up calls every 1–2 weeks to improve adherence), (2) both may alleviate non-CIPN symptoms (e.g., nausea), (3) both may have psychosocial benefits (e.g., increasing self-efficacy), and (4) we presented both with similar expectation of benefit using standardized talking points. We did not expect eating more fruits and vegetables would have large effects on CIPN based on prior work [21]. We assessed adherence and contamination using a Daily Diary of steps, minutes of resistance training, RPE, and servings of fruits and vegetables (Supplementary Figure 2).

### Adverse event (AE) monitoring.

We screened for AEs at least once every 2 weeks via patient interview and chart review and the referring oncologist characterized AEs using the NCI Common Terminology Criteria for Adverse Events (CTCAE) v4.0. Events graded 3–5 are reported.

### Brain MRI data processing.

**Figure 2:**
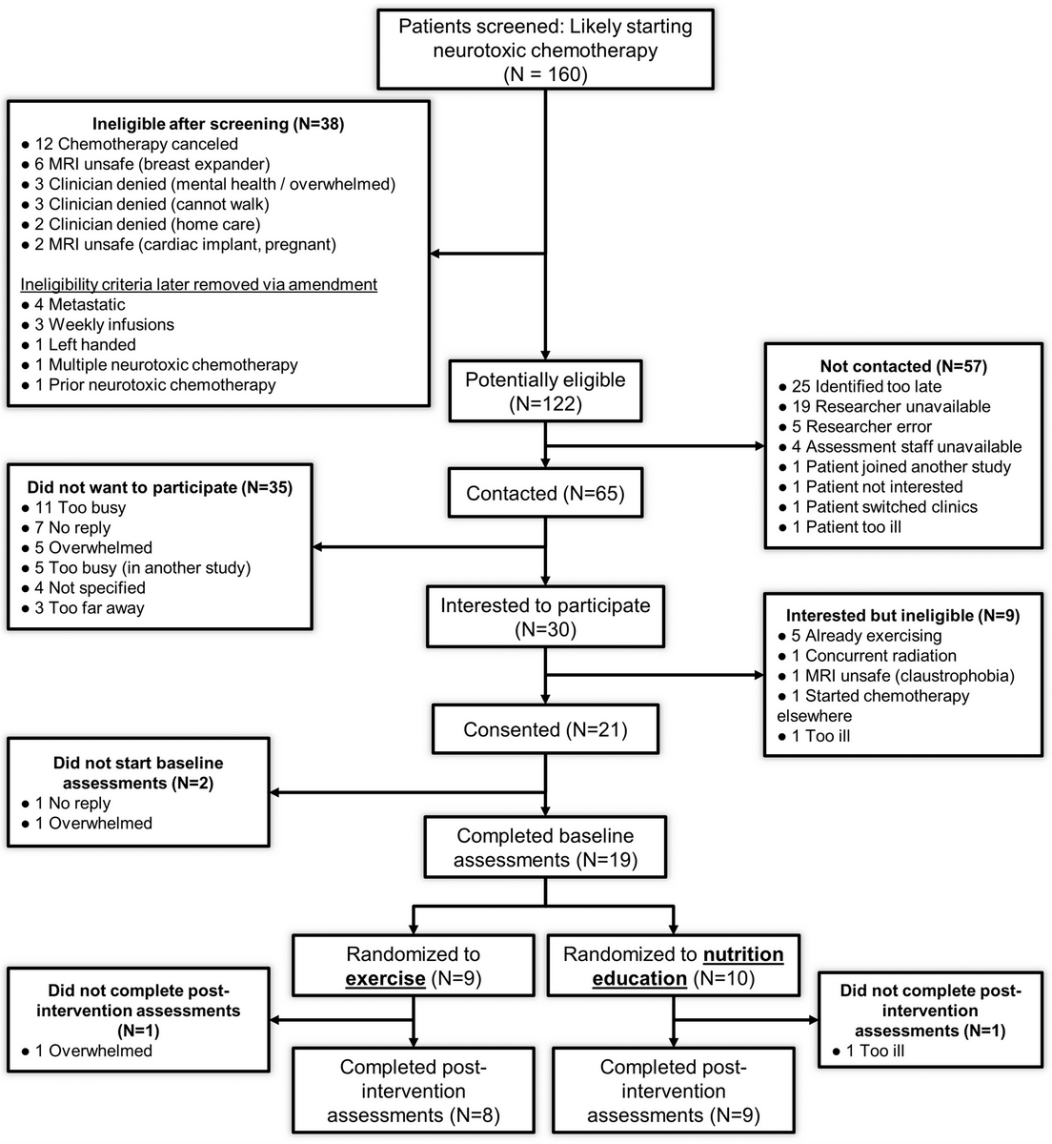
CONSORT diagram of screening, recruitment, and assessments.

All brain MRI data were processed and checked for quality using standard procedures in AFNI [22] and FreeSurfer [23]. The final dataset contained 26 resting fMRI scans (missing data are explained in Figure 2).

### Sample size and Statistical analyses.

We recruited as many participants as possible (up to 40) from summer 2017 to fall 2018 due to the timing of study funding, yielding 21 consents (2 withdrew before baseline).

All participants were analyzed in the group assigned via randomization. Analyses were performed using JMP (SAS Institute, Inc.; Cary, NC, USA) and MATLAB (Mathworks; Natick, MA). Due to the pilot nature of this work, we focused on standardized effect sizes (ES; not *p*-values). We calculated ES as Cohen’s *d* = (Mean difference) / (Pooled SD_pre_). To test the effects of exercise vs. control on an outcome, we used linear regression to model the outcome at mid- or post-intervention as a function of study arm, pre-intervention value of that outcome, and age because controls were older than exercisers due to random chance. To assess associations between brain fMRI measures and CIPN severity, we used linear regression to model CIPN-20 total score as a function of brain fMRI measure and age and subtracted that R^2^ by that of a null model of CIPN-20 as a function of age only; we only ran these analyses at T3 (after exposure to neurotoxic chemotherapy). For brain fMRI analyses, we imputed two missing values of CIPN-20 using a linear regression model across all participants: CIPN-20(post) = CIPN-20(pre) + Arm + Age.

## Results

### Participant recruitment (Figure 2) and characteristics (Table 1).

The nineteen participants spanned multiple cancer types and chemotherapy regimens. Dropouts were due to participants feeling overwhelmed or ill. Participants randomized to exercise were younger than controls (57 vs. 71 years; *p*=0.002) so we controlled for age in analyses comparing exercise vs. control. Participants received 10 ± 6 (mean ± SE) weeks of chemotherapy (range 3.9 – 31 weeks). By week 6, only 11% of participants (2 of 19) had completed chemotherapy whereas by week 12, 74% of participants (14 of 19) had completed chemotherapy (Supplemental Figure 3).

### Feasibility of data collection.

89% of participants (17 of 19) who completed baseline assessments completed the study. We conducted 86% of the planned monofilament touching tests (49 of 57) and 71% of the planned brain MRI scans (27 of 38). The missing data were due to a 6-week-long scanner upgrade and participants feeling ill or claustrophobic. For the fMRI data, 17 participants completed at least one MRI scan and 10 of these participants completed both MRI scans, yielding 27 scans, one of which had excessive head motion.

### Study acceptability.

At the end of the study, 95% of participants reported they would participate again. Participants reported enjoying study participation because: (1) it helped them focus on something positive during chemotherapy, (2) they wanted to help future patients, and (3) they enjoyed working with the study team. One of the exercise participants said “*I am pleased that I did it. You got me through some tough times. Forced me to get up and move even when I didn’t want to.*” One of the control participants said “*I think probably that the book with the food [the Nutrition Education book], it was helpful. It seems like very obvious content at first, but when you get that chemo brain fog, the book helps sort it out*.” The major criticisms of the study were the numerous assessments and appointments.

### Study safety.

There were 16 adverse events and all were deemed unrelated to study participation by the participant’s medical oncologist. There were no incidental findings from our brain MRI data.

### Exercise adherence and contamination.

By mid-intervention, exercisers performed more resistance exercise than controls: exercisers completed mean ± SE = 3.3 ± 2.1 (range 0.8 – 6.3) sessions/week with 86% of exercise participants (6/7) completing at least one session vs. controls 1.0 ± 1.7 (range 0 – 3.5) sessions/week with 25% (2/8) completing at least one session. Age-adjusted analyses estimated that exercisers performed 3.7 more sessions/week (*p*=0.118). For exercisers, resistance exercise sessions lasted on average 25.1 ± 10.3 min (range 5 – 38 min) at RPE 3.6 ± 0.6 (range 2.0 – 6.3). By post-intervention, exercisers again performed more resistance training than controls: 2.4 ± 0.8 (range 0.4 – 5.3) sessions/week vs. controls performing 0.7 ± 0.4 (range 0 – 1.8) sessions/week. Age-adjusted analyses estimated that exercisers performed 3.1 more sessions/week vs. controls (*p*=0.111). Resistance training sessions lasted on average 24.5 ± 4.1 min at RPE 3.7 ± 0.6 for exercisers.

For walking exercise, our age-adjusted models estimated no large differences in daily steps between groups. Specifically, at mid-intervention exercisers walked 725 steps/day more than controls (ES=0.33; *p*=0.621) and at post-intervention exercisers walked 715 steps/day less than controls (ES=0.30; *p*=0.726). When collapsing across groups, at pre-intervention participants walked 4283 ± 2414 steps on average, at mid-intervention this decreased to 3745 ± 1743 steps/day, and at post-intervention this increased back to near baseline at 4149 ± 2585 steps/day.

### Adherence to nutrition intervention (control condition).

Fruit and vegetables consumption remained similar throughout the study with no meaningful differences between groups (*p*>0.809). Specifically, exercisers went from 4.0 ± 0.6 servings/day at pre-intervention to 3.8 ± 0.6 by mid-intervention to 3.8 ± 0.4 by post-intervention. Control participants went from 3.3 ± 0.6 servings/day at pre-intervention to 3.7 ± 0.8 by mid-intervention to 3.8 ± 0.8 by post-intervention.

### Primary outcome: Effects of exercise on CIPN symptoms (Table 2, Figure 3).

At baseline, both exercisers and controls reported similar and mild levels of neurotoxicity after adjusting for age (CIPN-20 0–100 scores: 7.8 ± 3.7 and 8.8 ± 3.2 for exercisers and controls, respectively; Table 2 shows unadjusted values). At mid-intervention, both groups reported worse CIPN, but exercise attenuated the CIPN severity progression (age-adjusted CIPN-20: 14.8 ± 4.1 vs. 23.1 ± 3.8; ES = −0.85). Post-intervention was similar, wherein both groups reported worse CIPN and exercise attenuated the CIPN severity progression (age-adjusted CIPN-20: 17.3 ± 5.1 vs. 22.9 ± 5.1; ES = −0.51).

### Effects of exercise on other signs and symptoms of CIPN (Table 2).

Exercise attenuated patient-reported numbness and tingling, hot/coldness in hands/feet, and pain, typically with greater benefits at mid- vs. at post-intervention (ES values range −0.3 to −1.43; Table 2). For the monofilament tactile threshold outcomes, at mid intervention, exercise had beneficial effects on the left pointer finger pad (ES = −1.03) but not the right pointer finger pad (ES = −0.05). At post-intervention exercise had little to no effect on tactile threshold in the left pointer finger pad (ES = −0.06) and a detrimental effect on the right pointer finger pad (ES = 0.42). The other areas we tested were deemed feasible but not interpretable due to small sample sizes (they were added later in the study).

### Effects of exercise on physical function (Table 2).

Exercise improved all 8 measures of leg strength at mid-intervention (mean ES = 0.51, range 0.02 – 0.81) and had small/negligible improvements in 7 of the 8 measures at post-intervention (mean ES = 0.15, range −0.13 – 0.34), compared to control. In terms of handgrip strength, exercise showed small/negligible increases in strength on the right (ES = 0.17 and 0.16 at mid- and post-intervention) and small/negligible reductions on the left (ES = −0.18 and −0.23). For the 6-min walk test, exercise showed a small/negligible increase in distance at mid-intervention (ES = 0.22) and negligible reduction at post-intervention (ES = −0.16), compared to control.

### Association between CIPN severity and functional connectivity in the interoceptive brain system (Figure 4a-c).

**Figure 4:**
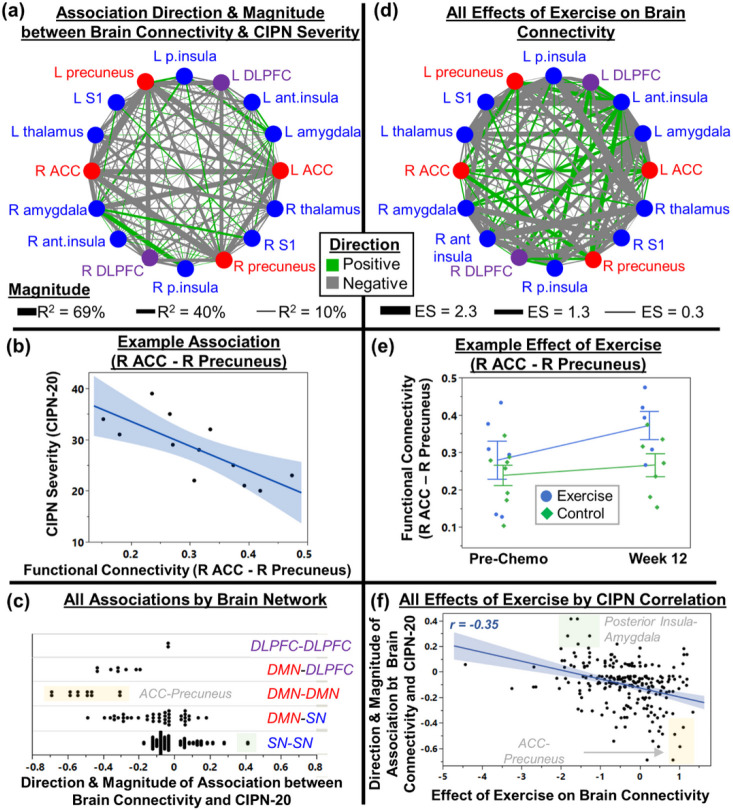
Results of brain fMRI analyses, suggesting that CIPN is related to brain connectivity and that exercise tended to mitigate the changes in brain connectivity values seen in more severe CIPN. (a) The network graph shows the association direction and magnitude between CIPN severity and functional connectivity between brain regions in the two networks of the interoceptive brain system: the default mode network (DMN, red) and the salience network (SN, blue), as well as the dorsolateral prefrontal cortex (DLPFC). We assessed the association direction from the regression coefficient (positive or negative) and we assessed magnitude using R^2^ from regression. (b) One example association between CIPN severity and brain connectivity (between the right anterior cingulate cortex [ACC] and right precuneus). (c) All associations between CIPN severity and brain connectivity separated by network, showing that within-DMN connectivity values are negatively associated with CIPN severity and within-SN connectivity values are positively associated with CIPN severity. (d) The network graph shows the effect of 12 weeks of exercise (vs. control) on brain connectivity values. (e) An example effect of exercise on brain connectivity (between the right ACC and right precuneus, same regions as in panel b). (f) Exercise tended to increase brain connectivity across regions whose connectivity values were negatively associated with CIPN severity (e.g., ACC – precuneus; yellow box in lower right) and exercise tended to decrease brain connectivity values across regions whose connectivity values were positively associated with CIPN severity (e.g., posterior insula – amygdala; green box in upper left).

First, we confirmed that the resting functional connectivity data from this sample comprised the interoceptive brain system with two networks (default mode network [DMN] and salience network [SN]), as seen in healthy adults [24]. Next, we explored whether CIPN symptom severity related to functional connectivity in the regions that comprise the DMN and SN. Patient-reported CIPN severity (CIPN-20) was associated with functional connectivity between several brain regions (Figure 4a). Many associations were negative (thick gray lines, example in Figure 4b). In Figure 4c, CIPN severity was negatively associated with functional connectivity between nodes of the DMN (e.g., anterior cingulate cortex [ACC]-precuneus, R^2^ = 40–69%), between DMN and dorsolateral prefrontal cortex (DLPFC; precuneus-DLPFC, ACC-DLPFC, R^2^ = 32–43%), and between DMN and SN (precuneus-thalamus, ACC-thalamus, ACC-amygdala, R^2^=34–49%). These negative associations suggest that patients with worse CIPN have weaker communication between these brain networks and regions, consistent with studies of other pain conditions including chronic back pain, chronic regional pain syndrome, and osteoarthritis [25, 26]. CIPN severity was positively associated with functional connectivity between nodes of the SN (e.g., amygdala-posterior insula, R^2^ = 41%). These positive associations suggest that patients with worse CIPN have stronger communication between brain regions within the SN, consistent with central sensitization seen in chronic pain [26]. Most of the regions we explored had connectivity that was not strongly associated with CIPN severity, so the strong associations shown here suggest areas of focus for future studies.

### Effects of exercise on functional connectivity in the interoceptive brain system (Figure 4d-f).

Exercise changed functional connectivity across several nodes of the interoceptive brain system (Figure 4d; example in Figure 4e). Exercise increased connectivity values that were lower in participants with worse CIPN (e.g., ACC-precuneus, yellow box at the bottom right of Figure 4f). Also, exercise decreased connectivity values that were higher in participants with worse CIPN (e.g., posterior insula-amygdala, green box at the top of Figure 4f). Exercise tended to decrease functional connectivity values between the nodes that we explored (mean ± SD ES = −0.36 ± 0.88, *p*<0.0001) and did not have large effects on a third of the values tested (94/289 have |ES| < 0.2). Taken together, it appears that exercise changed brain functional connectivity values across several regions, moving the connectivity values closer towards those seen in patients with less severe CIPN.

## Discussion

Our results suggest the feasibility and acceptability of our RCT comparing home-based walking and resistance exercise to an active behavioral control condition (nutrition education) during neurotoxic chemotherapy while assessing CIPN symptoms, CIPN signs, physical function, and the interoceptive brain system using fMRI. Our data also suggest that exercise attenuated CIPN symptoms and improved strength, with clinically meaningful improvements in CIPN-20 scores of −7.9 ± 5.7 points after 6 weeks and −4.8 ± 7.3 points after 12 weeks (minimum clinically important difference is 5–6 points [19]). We observed a greater benefit of exercise on CIPN and other outcomes at 6 vs. 12 weeks. This is perhaps because many participants completed their chemotherapy between 6 and 12 weeks, and exercise may help most during active chemotherapy treatment when patients are more likely to further reduce physical activity in the absence of a structured exercise program. Our fMRI results suggest that changes in functional connectivity within the interoceptive brain system play a role in CIPN and its treatment via exercise.

This is the first study to assess brain function in an RCT of exercise for CIPN, which can help us optimize potential beneficial effects of exercise for CIPN [16]. This is also the first study of exercise for CIPN using a behavioral placebo condition to our knowledge (most studies use usual care control [27]). A behavioral placebo is important to control for non-specific factors and to reveal whether outcomes are affected by exercise-specific factors [20] (e.g., neuromuscular and systemic effects of muscle contraction and aerobic activity and downstream biopsychosocial effects [16]).

Our findings suggest the brain is involved in CIPN and that exercise can affect the brain via avenues that can attenuate the progression of CIPN. Specifically, our results suggest that the strongest associations with CIPN severity involve decreased functional connectivity within the DMN. The DMN supports a wide range of functions (see Figure 5 of [24]), and we hypothesize that the DMN helps maintain and update the brain’s model of the body, shaping our perceptions including symptoms [24]. We also found that CIPN severity is worse with lower DMN-DLPFC connectivity, suggesting less effective descending pain inhibition [28]. These results suggest that CIPN symptoms may result not just from peripheral nerve damage but also from how the brain represents the body (DMN) and how the brain modulates incoming peripheral signals (DMN-DLPFC). We also found that exercise increases DMN connectivity while it ameliorates CIPN symptoms, which was associated with reduced DMN connectivity.

These findings of the role of the brain in CIPN invite two hypotheses: (1) we can measure CIPN risk by measuring the brain, as suggested by others e.g., [29], and (2) interventions that change the brain’s representation of the body can prevent or treat CIPN (e.g., exercise, meditation, psychotherapy, certain pharmaceuticals). However, we do not know whether these changes in brain connectivity are consistent and specific to CIPN, as the brain is notoriously complex with many redundant mechanisms that support subjective experience including symptoms—future studies with larger sample sizes can compare how these brain measures are associated with CIPN vs. other symptoms such as fatigue, anxiety, and depression, which are strongly associated with CIPN [30]. In addition, the changes in network- and region-level functional connectivity that we observed here may result from a lower-level mechanisms including molecular changes (e.g., inflammation, neurotransmitters such as γ-aminobutyric acid [GABA]) and cellular changes (e.g., synaptic connections, gray matter density), which may be targetable with drugs or other interventions [14].

This work has several clinical implications. First, if exercise can alleviate CIPN, it should be started as early as possible, per our recent framework suggesting how physical therapists can help assess and treat CIPN [31]. In the far future, we hope to leverage brain measures to help predict CIPN and its chronicity [14]; we are actively developing new brain fMRI outcomes to assess CIPN (e.g., by having participants think about the site of their symptoms [15]) and we are trying to identify who might benefit most from exercise and how to optimize its effects [16]. In the future, exercise could complement proven CIPN therapeutics such as duloxetine, alongside other promising yet unproven therapeutics such as cryocompression, electrical nerve stimulation, and acupuncture [6, 10].

This study has several key strengths. First, the design is rigorous, including randomization, three time points, several CIPN assessments (per recent guidelines CIPN assessment in exercise studies [32]), and the use of a time- and attention-matched behavioral control condition: nutrition education. Second, we used brain fMRI to study both CIPN and the effects of exercise using a novel perspective of the role of the brain in CIPN [14]. The brain MRI scans we chose utilize one of the most widely used and well validated measures to the study the human brain—resting functional connectivity [33].

This study also has a few limitations. First, the sample size is relatively small (*n*=19), but this is common for pilot studies (e.g., 10–14 participants [34–36]). Second, there is heterogeneity in cancer and chemotherapy types (Table 1), but this helped us learn where to best focus our efforts next: namely breast and gastrointestinal cancer. Third, exercise adherence was not very good for the daily walking exercises, with exercisers walking less than controls at 12 weeks. However, the adherence was good for resistance exercise, and we are trying to increase adherence in future trials by studying predictors of daily exercise behavior [37]. Moreover, contamination of exercise by control participants showed that the minority of participants who did exercise did so for longer than those randomized to exercise, but the exercise was not consistently done week after week as it was in the exercisers. Next, the fact that the exercise participants were younger due to random chance may have confounded the effects of exercise on CIPN if younger patients have less severe CIPN, but the link between CIPN and age is mixed (see [38]) and we controlled for age analytically. Finally, there may be residual confounding in any of the correlational findings between CIPN and the brain, and we do not know the extent to which changes in the brain contribute to CIPN symptoms vs. CIPN symptoms contribute to changes in the brain, as our prior review provides evidence for both causal directions [14].

In conclusion, our results suggest the feasibility and acceptability of an RCT testing home-based walking and resistance exercise vs. time- and attention-matched behavioral placebo during neurotoxic chemotherapy to measure CIPN symptoms, signs, physical function, and brain-based mechanisms. Our small dataset also tentatively suggests that exercise during neurotoxic chemotherapy can partially protect against CIPN with clinically meaningful benefits, and that the interoceptive brain system plays a role in CIPN and its treatment by exercise. We are currently testing for replication and extension with three Phase II RCTs (NCT03858153, NCT05452902, and NCT04888988).

## Figures and Tables

**Figure 3:**
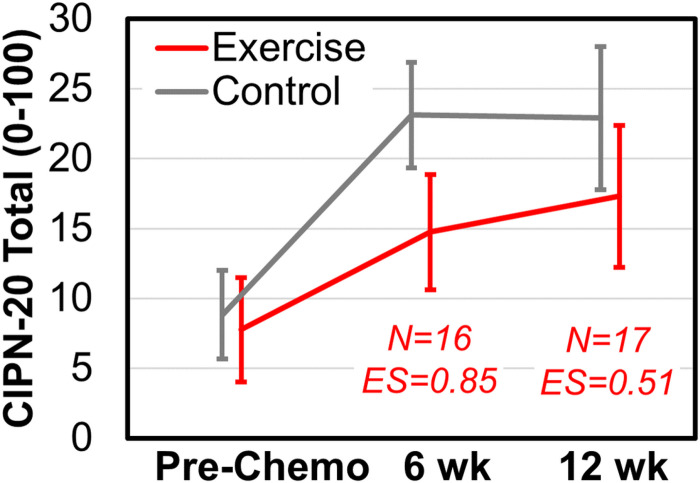
Exercise may attenuate the severity of chemotherapy-induced peripheral neurotoxicity (CIPN) rated by the CIPN-20, compared to nutrition education control. Error bars correspond to standard errors. ES is effect size using Cohen’s d. These are age- and pre-intervention-adjusted values. Unadjusted values are in Table 2. Scores range from 0–100 and higher is worse CIPN.

**Table 1.** Baseline characteristics

Characteristic	Exercise	Control (Nutrition Education)	Total	Test for Difference Between Control and Exercise	
				Test^a^	*p*
**Total participants**	9	10	19		
**Female sex**	5	5	10	*FE*	0.809
**Age, years (mean ± SD)**	57.1 ± 7.8	71.1 ± 7.8	64.6 ± 10.6	t	0.002
**Race**				*FE*	
White	8	10	18		0.473
Non-White	1	0	1		
**Employment**				*FE*	0.400
Employed outside the house	2	1	3		
Self-employed / homemaker	1	0	1		
Unemployed	0	2	2		
Not reported	6	7	13		
**Marital status**				*FE*	0.181
Married or long-term committed relationship	6	3	9		
Divorced, separated, single, widowed	0	3	3		
Not reported	3	4	7		
**Education**				*FE*	0.649
At least some college	4	4	8		
High school/GED degree	2	1	3		
No high school or GED degree	0	0	0		
Not reported	3	5	8		
**Cancer type**				*FE*	0.637
Bladder	1	0	1		
Breast	4	4	8		
Colon	2	1	3		
Esophageal	0	1	1		
Myeloma	1	2	3		
Pancreatic	1	1	2		
Prostate	0	1	1		
**Neurotoxic chemotherapy type**				*FE*	1.00
Bortezomib	1	2	3		
Cisplatin + Vinblastine	1	0	1		
Docetaxel	0	1	1		
Oxaliplatin	3	2	5		
Paclitaxel	4	5	9		

a Statistical tests includes t-test or Fisher’s exact (FE) test with χ^2^ likelihood ratio statistic

GED, general educational development

**Table 2.** Results for key study outcomes with age-adjusted regression analyses

Outcome	Exercise			Behavioral Placebo Control			Exercise minus Control ^a^					
	Pre ^b^ *Mean ± SE (num. observations)*	Mid ^b^	Post ^b^	Pre ^b^	Mid ^b^	Post ^b^	Mid - Pre			Post - Pre		
							Estimated difference ^a^	Effect Size ^c^	p-value	Estimated difference ^a^	Effect Size	p-value
**CIPN signs and symptoms (higher is worse)**												
CIPN-20 Total (range 0–100)	5.1 ± 2.5 (9)	16.3 ± 4.0 (7)	18.6 ± 6.0 (8)	11.2 ± 3.2 (10)	21.9 ± 2.5 (9)	20.8 ± 2.7 (9)	−7.9 ± 5.7	−0.85	0.19	−4.8 ± 7.3	−0.51	0.53
CIPN-20 Sensory (range 0–100)	5.3 ± 3.1 (9)	18.5 ± 5.0 (7)	23.2 ± 7.7 (8)	10.0 ± 3.1 (10)	21.0 ± 3.9 (9)	22.2 ± 4.6 (9)	−10.5 ± 7.2	−0.81	0.17	−6.0 ± 8.6	−0.46	0.50
CIPN-20 Motor (range 0–100)	4.2 ± 2.7 (9)	11.3 ± 5.0 (7)	12.5 ± 5.0 (8)	7.5 ± 3.4 (10)	19.4 ± 3.7 (9)	13.4 ± 2.4 (9)	−10.1 ± 8.5	−0.79	0.26	−5.8 ± 6.2	−0.46	0.37
CIPN-20 Autonomic (range 0–100)	6.8 ± 3.9 (9)	25.4 ± 10.4 (7)	22.2 ± 8.4 (8)	25.0 ± 6.7 (10)	30.2 ± 7.2 (9)	35.2 ± 9.5 (9)	11.2 ± 15.3	0.51	0.48	2.7 ± 15.1	0.12	0.86
Patient-reported numbness/tingling (range 0–10)	0.6 ± 0.4 (8)	2.4 ± 0.9 (7)	1.0 ± 0.4 (5)	1.1 ± 0.7 (9)	2.4 ± 0.9 (7)	3.7 ± 1.0 (6)	−1.2 ± 1.3	−0.77	0.37	−1.5 ± 0.9	−0.91	0.16
Patient-reported hot/coldness in hands/feet (range 0–10)	1.3 ± 1.0 (8)	0.9 ± 0.3 (7)	0.0 ± 0.0 (5)	1.1 ± 0.6 (10)	4.0 ± 1.3 (7)	2.0 ± 1.1 (5)	−0.7 ± 1.7	−0.30	0.71	−0.7 ± 1.7	−0.29	0.71
Patient-reported pain (range 0–10)	3.3 ± 1.1 (8)	4.3 ± 1.8 (7)	1.0 ± 0.8 (5)	1.7 ± 0.4 (10)	3.7 ± 1.2 (6)	3.3 ± 1.3 (6)	−3.5 ± 2.5	−1.43	0.20	−2.3 ± 2.3	−0.95	0.35
Tactile Threshold: Left pointer finger pad (ln g)	−2.8 ± 0.3 (9)	−3.1 ± 0.1 (7)	−3.1 ± 0.2 (8)	−2.9 ± 0.2 (10)	−2.6 ± 0.2 (7)	−2.8 ± 0.2 (8)	−0.8 ± 0.3	−1.03	0.03	0.0 ± 0.3	−0.06	0.88
Tactile Threshold: Right pointer finger pad (ln g)	−2.4 ± 0.4 (9)	−2.7 ± 0.2 (7)	−2.4 ± 0.4 (8)	−2.1 ± 0.3 (10)	−2.4 ± 0.3 (7)	−2.4 ± 0.3 (8)	0.0 ± 0.5	−0.05	0.93	0.4 ± 0.6	0.42	0.48
**Physical function (higher is better)**												
Leg Strength: Right Knee Extension (peak torque, ft·lbs)	80.6 ± 9.8 (7)	78.3 ± 9.7 (6)	78.3 ± 8.6 (6)	70.8 ± 4.7 (8)	69.7 ± 7.7 (6)	72.4 ± 7.4 (6)	0.4 ± 9.0	0.02	0.96	4.2 ± 9.2	0.21	0.66
Leg Strength: Right Knee Extension (average power, watts)	63.9 ± 7.8 (7)	64.4 ± 7.7 (6)	65.8 ± 7.0 (6)	56.9 ± 4.4 (8)	58.5 ± 6.2 (6)	58.7 ± 7.0 (6)	3.4 ± 8.3	0.21	0.69	5.1 ± 8.7	0.31	0.58
Leg Strength: Left Knee Extension (peak torque, ft·lbs)	77.8 ± 13.9 (7)	76.8 ± 11.3 (6)	77.3 ± 9.5 (6)	59.4 ± 7.0 (8)	65.7 ± 6.5 (6)	69.3 ± 8.4 (6)	10.4 ± 7.1	0.35	0.18	8.1 ± 8.6	0.27	0.38
Leg Strength: Left Knee Extension (average power, watts)	59.4 ± 10.4 (7)	65.4 ± 11.2 (6)	66.7 ± 8.3 (6)	45.9 ± 5.2 (8)	52.5 ± 4.8 (6)	59.0 ± 7.5 (6)	9.3 ± 6.6	0.42	0.19	7.6 ± 7.1	0.34	0.32
Leg Strength: Right Knee Flexion (peak torque, ft·lbs)	34.2 ± 5.8 (7)	35.9 ± 5.2 (6)	33.0 ± 4.5 (6)	33.1 ± 4.0 (8)	32.0 ± 3.6 (6)	34.1 ± 3.8 (6)	9.5 ± 4.7	0.74	0.08	1.6 ± 5.1	0.13	0.76
Leg Strength: Right Knee Flexion (average power, watts)	27.3 ± 5.5 (7)	30.5 ± 5.0 (6)	29.3 ± 4.1 (6)	25.4 ± 4.3 (8)	27.0 ± 2.7 (6)	29.7 ± 3.9 (6)	9.2 ± 3.8	0.71	0.04	1.1 ± 5.0	0.08	0.84
Leg Strength: Left Knee Flexion (peak torque, ft·lbs)	31.8 ± 5.9 (7)	34.7 ± 5.1 (6)	34.2 ± 4.5 (6)	30.4 ± 3.1 (8)	31.1 ± 4.5 (6)	35.7 ± 4.9 (6)	9.8 ± 6.0	0.81	0.14	0.0 ± 7.1	0.00	1.00
Leg Strength: Left Knee Flexion (average power, watts)	23.5 ± 5.5 (7)	27.5 ± 5.0 (6)	30.2 ± 4.2 (6)	22.5 ± 4.0 (8)	24.9 ± 4.0 (6)	32.1 ± 5.0 (6)	9.8 ± 3.9	0.79	0.04	−1.6 ± 7.9	−0.13	0.84
Handgrip strength: Right (kg)	28.3 ± 2.9 (8)	28.5 ± 3.0 (7)	27.5 ± 2.8 (8)	26.6 ± 2.6 (9)	27.8 ± 3.2 (6)	26.5 ± 3.6 (7)	1.3 ± 1.4	0.17	0.37	1.2 ± 1.8	0.16	0.50
Handgrip strength: Left (kg)	30.0 ± 2.6 (8)	30.8 ± 3.3 (7)	28.0 ± 2.5 (8)	28.1 ± 2.2 (9)	27.7 ± 3.3 (6)	27.2 ± 3.5 (7)	−1.2 ± 1.4	−0.18	0.39	−1.6 ± 2.7	−0.23	0.57
Six Minute Walk Test Distance (feet)	1731 ± 137 (7)	1784 ± 157 (5)	1746 ± 159 (5)	1609 ± 90 (7)	1689 ± 109 (5)	1728 ± 80 (6)	66 ± 122	0.22	0.61	−48 ± 97	−0.16	0.64
**Exercise adherence and contamination**												
Daily Steps	4221.1 ± 853.9 (8)	3683.5 ± 846.7 (7)	4309.7 ± 1072.0 (7)	4345.4 ± 911.3 (8)	3817.0 ± 457.3 (6)	3923.8 ± 1114.0 (5)	787.1 ± 1537.7	0.33	0.62	−725.6 ± 2003.1	−0.30	0.73
Cumulative number of resistance workouts	0.8 ± 0.5 (5)	19.5 ± 5.1 (6)	29.0 ± 9.4 (6)	0.0 ± 0.0 (2)	6.0 ± 5.0 (4)	8.0 ± 5.0 (4)	45.2 ± 8.5	46.34	0.12	74.0 ± 13.1	75.83	0.11
Duration of resistance workouts (minutes)	26.5 ± 11.5 (2)	25.1 ± 4.2 (6)	24.5 ± 4.1 (6)	-	52.6 ± 31.0 (2)	69.3 ± 14.3 (2)	-	-	-	-	-	-
Rating of perceived exertion (RPE) of resistance workouts	2.5 ± 0.5 (2)	3.6 ± 0.6 (6)	3.7 ± 0.6 (6)	-	3.4 ± 0.6 (2)	3.6 ± 0.8 (2)	-	-	-	-	-	-
**Control intervention adherence**												
Daily servings of fruits and vegetables	4.0 ± 0.6 (7)	3.8 ± 0.6 (7)	3.8 ± 0.4 (7)	3.3 ± 0.6 (6)	3.7 ± 0.8 (8)	3.8 ± 0.8 (8)	0.2 ± 1.5	0.11	0.92	0.3 ± 1.6	0.21	0.85

a Separate models were used to analyze data at T2 and T3, each controlling for data at pre-intervention and age because control participants were older due to random chance. Estimated difference shows the “arm” coefficient (exercise vs. control).

b mean ± SE (number of observations)

c Effect size is calculated as model-estimated effect of exercise divided by baseline standard deviation. Positive effect sizes show that exercise increased the outcome
